# First person – Lana de Vries

**DOI:** 10.1242/bio.053413

**Published:** 2020-06-23

## Abstract

First Person is a series of interviews with the first authors of a selection of papers published in Biology Open, helping early-career researchers promote themselves alongside their papers. Lana de Vries is first author on ‘Bumblebees land remarkably well in red–blue greenhouse LED light conditions’, published in BiO. Lana is a PhD Candidate in the lab of Dr Florian Muijres at the Experimental Zoology Group, Wageningen University & Research, studying insects, especially social insects like bumblebees, since these kinds of animals can build complex social systems with an organized division of tasks.


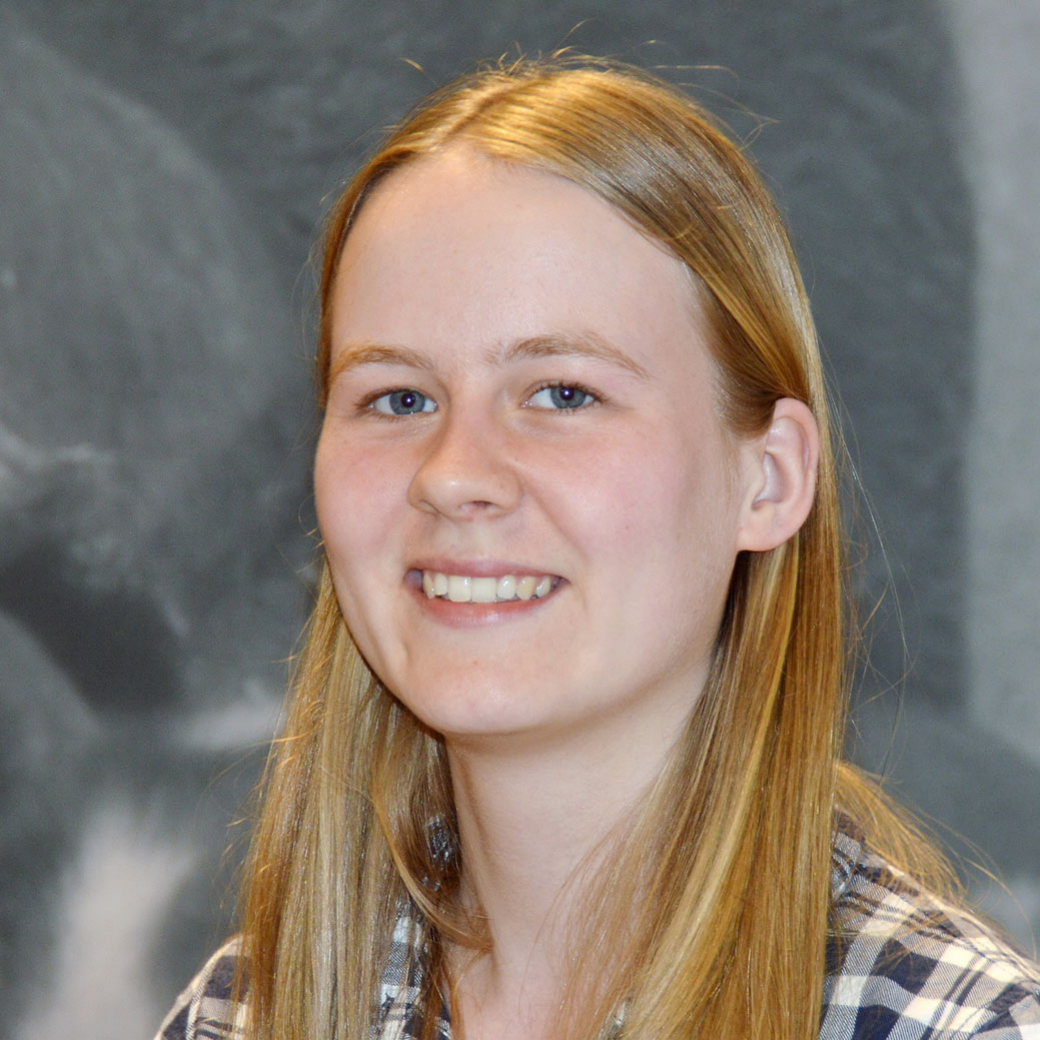


**Lana de Vries**

**What is your scientific background and the general focus of your lab?**

I studied biology with a focus on ecology. During my studies, I developed an interest in both plants and insects. However, during my masters program, I decided in the end I would prefer working on insects, since insect experiments are more dynamic than experiments with plants. Now, during my PhD project, I am studying the visual–motor system of bumblebees, by filming landing bumblebees using high-speed videography. Our lab, the Experimental Zoology Group at Wageningen University & Research, focusses on studying the functional zoology of animals. A broad range of animals are being studied, including for example bumblebees, mosquitoes, tree frogs, live bearing fish and zebrafish.

“I am studying the visual–motor system of bumblebees, by filming landing bumblebees using high-speed videography.”

**How would you explain the main findings of your paper to non-scientific family and friends?**

In the last few decades, greenhouses have been experimenting with using red–blue light conditions to improve plant growth. We studied the effect of these red–blue light conditions on the landing ability of bumblebees, which are often used inside greenhouses to pollinate plants. Bumblebees can hardly see red light, as their visual system is most sensitive to green light. Therefore, we expected the landing ability of bumblebees would be negatively affected by the red–blue light conditions. Surprisingly, we found the landing ability of bumblebees is only slightly affected by the red–blue light spectrum. Only the final part of the landing, after the bumblebee has extended its leg, took 0.05 s (25%) longer in red–blue light than in white light. We did not find an effect on the duration of the total landing. So, although we did find an effect of the red–blue light spectrum on the landing ability of bumblebees, our research showed that bumblebees can still land remarkably well in these light conditions.

**What are the potential implications of these results for your field of research?**

I think our study shows that bumblebees can deal surprisingly well with large changes in the light conditions. We found that their landing ability was still remarkably good in red–blue lighting. However, our study is only a starting point in the research that is needed to provide information on the effect of changed light conditions inside greenhouses on species used for pollination or biological control. For example, orientation ability and flower search behavior could be affected as well. Clearly more research on the impacts of spectral changes in the light inside greenhouses is needed.

**What has surprised you the most while conducting your research?**

While conducting my research, the flexibility and learning ability of bumblebees has surprised me quite a lot. If you want to, and you do it step by step, you can train them to do many things. For example, as preparation for experiments, I trained the worker bumblebees of a hive to fly across a 1 by 1 m flight box, go through a hole in the wall and walk through a 20 cm tube containing two one-way doors to get to a food source. To get back to the flight box, they had to take a different exit and walk through a 60 cm tube containing two more one-way doors.

“While conducting my research, the flexibility and learning ability of bumblebees has surprised me quite a lot.”

**What, in your opinion, are some of the greatest achievements in your field and how has this influenced your research?**

The development of efficient blue LEDs has been a large advance in technology; without this invention, I may have been working on a different research topic. Green and red LEDs were already available for some decades, but the invention of an efficient blue LED by Shuji Nakamura made it possible to manipulate the light spectrum with great precision. Among many other applications, this made it possible for greenhouses to optimize their light spectrum for plant growth. We studied the effects of these changes in the light spectrum on landing behavior of bumblebees, which are often used for pollination inside greenhouses. During the rest of my PhD project, I will continue using narrow spectrum LEDs to help me answer my research questions on the visual-motor system of bumblebees.

**What changes do you think could improve the professional lives of early-career scientists?**

Currently, it is difficult as an early-career scientist to get a permanent position. Scientists are expected to do several postdocs before you they can enter a tenure track position, and even then, it is not certain they can stay. As a result, it seems not the best time to buy a house or start a family. I think this could be improved, it should be easier to get a permanent position as an early-career scientist, without having to spend years in temporary positions.

**What's next for you?**

First, I still have two and a half years to go in my PhD project. After finishing my PhD thesis, I would like to stay in academia, since I like performing research and teaching at a university. It would be nice if I could continue working on insect behavior or ecology. I am thinking about writing my own research proposal for a postdoc, since at some point I think you need to be able to get research funding if you want to stay in academia.
